# The Role of Vitamin D Oral Supplementation in Insulin Resistance in Women with Polycystic Ovary Syndrome: A Systematic Review and Meta-Analysis of Randomized Controlled Trials

**DOI:** 10.3390/nu10111637

**Published:** 2018-11-02

**Authors:** Karolina Łagowska, Joanna Bajerska, Małgorzata Jamka

**Affiliations:** 1 Institute ofHuman Nutrition and Dietetics, Poznań University of Life Sciences, Wojska Polskiego 31, 60-624 Poznań, Poland; joanna.bajerska@up.poznan.pl; 2 Department of Pediatric Gastroenterology and Metabolic Diseases, Poznań University of Medical Sciences, Szpitalna 27/33, 60-572 Poznań, Poland; m_jamka@wp.pl

**Keywords:** vitamin D, 25(OH)D, insulin resistant, glucose, polycystic ovary syndrome

## Abstract

Objective: To evaluate the effect of vitamin D supplementation (alone or with co-supplementation) on insulin resistance in patients with polycystic ovary syndrome (PCOS). Methods: We performed a literature search of databases (Medline, Scopus, Web of Knowledge, Cochrane Library) and identified all reports of randomized controlled trials (RCTs) published prior to April 2018. We compared the effects of supplementation with vitamin D alone (dose from 1000 IU/d to 60,000 IU/week) or with co-supplements to the administration of placebos in women diagnosed with PCOS. The systematic review and meta-analysis protocol was registered in the International Prospective Register of Systematic Reviews (Prospero) as number CRD42018090572. Main results: Eleven of 345 identified studies were included in the analysis; these involved 601women diagnosed with PCOS. Vitamin D as a co-supplement was found to significantly decrease fasting glucose concentrations and the HOMA-IR value. HOMA-IR also declined significantly when vitamin D was supplemented with a dose lower than 4000 IU/d. Conclusions: Evidence from RCTs suggests that the supplementation of PCOS patients with continuous low doses of vitamin D (<4000 IU/d) or supplementation with vitamin D as a co-supplement may improve insulin sensitivity in terms of the fasting glucose concentration (supplementation with vitamin D in combination with other micronutrients) and HOMA-IR (supplementation with vitamin D in continuous low daily doses or as co-supplement).

## 1. Introduction

Polycystic ovary syndrome (PCOS) is a common endocrine disorder frequently diagnosed among women of reproductive age, and has a prevalence of 5–20% [1,2]. The 2003 Rotterdam diagnostic criteria for PCOS are based on the presence of at least two of the following criteria: irregular or lack of ovulation, increased levels of androgen hormones, and enlarged ovaries containing at least 12 follicles each. As many as 50 to 70% of women with PCOS are insulin resistant [3]. Most of the evidence shows that insulin resistance appears to potentiate excess androgen production in adolescent and adult PCOS patients. It has been repeatedly indicted that treatment of insulin resistance leads to an improvement in reproductive and metabolic abnormalities, and probably reduces the future risk of developing diabetes and cardiovascular disease in PCOS women [4,5,6,7]. Thus, every action that might reduce hyperinsulinemia and its consequences in PCOS patients should be taken into consideration [5].

So far, the first line of treatment for women with PCOS and insulin resistance has been the administration of an antihyperglycemic agent called metformin. Numerous studies in adults have shown the favorable effect of metformin on glucose tolerance in type-2 diabetes, and other data also indicate that metformin is an effective agent in reducing insulin resistance and hyperandrogenism in adult women with PCOS [8]. However, it has been observed that metformin is also often poorly tolerated because of gastrointestinal side effects, such as nausea (61%), vomiting (30%), and diarrhea (65%) [9,10]. New economical and safe therapeutic approaches to treating insulin resistance in women with PCOS are therefore needed. To this end, the role of vitamin D in the regulation of insulin secretion has recently attracted much attention. It has been suggested that vitamin D supplementation could affect insulin secretion and improve glucose homeostasis in obese people with type-2 diabetes [11,12,13,14]. Low levels of 25-hydroxycholecalicferol (25(OH)D) are also commonly seen in women suffering from both PCOS and insulin resistance [15,16,17,18,19,20,21,22,23,24]. Several studies on PCOS have demonstrated that serum 25-hydroxyvitamin D (25OHD) concentrations are negatively correlated with body mass index (BMI), body fat, and insulin resistance [25,26]. However, the effects of vitamin D supplementation on insulin resistance treatment in women with PCOS are ambiguous.

We thus wish to answer the following question: can oral supplementation of vitamin D, alone or as a co-supplement, be beneficial in the treatment of insulin resistance in women with PCOS?

The aim of this study was to assess the effect of vitamin D supplementation on insulin resistance in women with PCOS, based on data available in randomized controlled trials.

## 2. Methods

### 2.1. Protocol and Registration

Our systematic review and meta-analysis protocol was registered in the International Prospective Register of Systematic Reviews (Prospero) as number CRD42018090572 [27]. The systematic review and meta-analysis process and manuscript development are consistent with the PRISMA guidelines [28].

### 2.2. Eligibility Criteria

Potential studies were identified from February to April 2018 by conducting a systematic search using PubMed (Medline), Scopus, Web of Science, and the Cochrane Library. During this process, we identified publications that describe the effect of vitamin D supplementation (alone or as a co-supplement) on insulin resistance in women with PCOS, without any limitation on the date of study publication or age of study participants.

The following index terms were used: vitamin D OR ergocalciferol OR cholecalciferol OR 25-hydroxyvitamin D 2 OR hydroxycholecalciferol OR calcifediol OR calcitriol OR 24,25-dihydroxyvitamin D 3 OR dihydrotachysterol AND polycystic ovary syndrome OR PCOS AND OR insulin resistance OR diabetes OR fasting insulin OR fasting glucose OR oral glucose test OR OGTT OR homeostasis model assessment ORHOMA-IR OR quantitative insulin sensitivity check index ORQUICKI OR fasting insulin resistance index ORFIRI OR insulinogenic index ORIGI OR hemoglobin A1c OR glycosylated hemoglobin ORHbA1c.

In addition, we also scanned the reference lists of the retrieved articles to identify additional relevant studies. The date of the final search was 30 April 2018.

### 2.3. Inclusion Criteria

The following were the inclusion criteria:

1. Intervention studies (randomized controlled trials (RCTs) and double-blind randomized controlled trials (DB-RCTs)) that compared vitamin D supplementation (alone or in combination with other vitamins and minerals) with a placebo without any limitations on time of supplementation.
2. English-language articles.
3. Studies that enrolled women with a strict diagnosis of PCOS using the Rotterdam criteria of the European Society of Human Reproduction and Embryology (ESHRE)/American Society of Reproductive Medicine (ASRAM) [29].
4. The vitamin D was administered orally as cholecalciferol (vitamin D3), ergocalciferol (vitamin D2), or an active form of vitamin D (1α-hydroxyvitamin D and 1,25-dihydroxyvitamin D (1,25(OH)2D)). Studies in which vitamin D was combined with other vitamins and minerals were also taken into consideration. Finally, we included studies that reported the most often assessed parameters in insulin resistance, such as fasting glucose, fasting insulin, and HOMA-IR.

### 2.4. Exclusion Criteria

The exclusion criteria were as follows:

1. Studies performed in specific groups of patients (e.g., subjects with hyperparathyroidism, subjects suffering from hepatic disease or kidney disease, those with a history of bariatric surgery, pregnant or breast-feeding women, and studies performed in women with PCOS but without insulin resistance).
2. Studies in which vitamin D was combined with metformin.
3. Conference papers, and articles only available in abstract form (where the authors could not be contacted) were also excluded.

### 2.5. Data Extraction and Analysis

The data was extracted independently by two reviewers (J.B. and M.J.) based upon the exclusion and inclusion criteria. Publications were assessed according to their titles, abstracts, and full texts in subsequent stages. Doubts were resolved by consensus between reviewers. Each selected publication was studied critically. If the publications included for full-text analysis were not available in the full version, the authors were contacted directly. To assess the study quality, a nine-point scoring system with the Newcastle–Ottawa scale was used. The top score was 9, and a high-quality study was defined by a threshold of at least seven points [30]. 

Two of the authors (K.Ł., J.B.) independently evaluated the eligibility of all retrieved studies from the databases and extracted the relevant data from each included study. During the data abstraction process, no attempt was made to contact the authors for further information beyond what had been published. Disagreements were resolved by consensus or arbitration. We abstracted the following data from each study: the first author’s name; year of publication; journal name; country; study design; sample size; diagnostic criteria for PCOS; full descriptions of participants enrolled (age, weight, and BMI); the interventions used (type and frequency); the placebo interventions; and the main outcomes—such as serum vitamin D, fasting glucose, fasting insulin, and HOMA-IR.

In our meta-analysis, results from RCTs were also classified by type of dose—low (<4000 IU/d vitamin D) or high dose (otherwise); supplementation frequency (daily or weekly); and type of supplementation (vitamin D alone or as a co-supplement in combination with calcium, vitamin K, zinc, or magnesium). 

In assessing the changes in serum 25(OH)D concentrations following vitamin D supplementation, the recommendations of the Endocrine Society were used, as they can be applied to both the general population and groups at risk of vitamin D deficiency [31]. Vitamin D deficiency was defined as a serum 25(OH)D concentrationof20–30 ng/mL, insufficiency as <20 ng/mL, and sufficiency as 30–80ng/mL. However, according to Sempos et al. [32], 25(OH)D values below 12 ng/mL should be considered as being associated with an increased risk of rickets or osteomalacia, while 25(OH)D concentrations between 20 ng/mL and 50 ng/mL appear to be safe and sufficient. The recommendations of the American Diabetes Association (ADA) were used to assess fasting plasma glucose and insulin concentrations [33]. Impaired glucose tolerance (IGT) was defined as a fasting plasma glucose concentration of 100–125 mg/dL. A reference range for fasting insulin of <25 μLU/mL was assumed. The changes in the HOMA-IR index during supplementation were used to assess the alterations in insulin resistance within the studied populations. Following ATP III-Met (Adult Treatment Panel III and the Metabolic Equivalent of Task), we defined the cut-off values of HOMA-IR for the diagnosis of insulin resistance as ≥1.8 [34]. 

### 2.6. Assessment of the Risk of Bias in Studies

To assess the methodological quality of the included RCTs, we used the Cochrane risk-of-bias assessment tool to evaluate the randomization performance and methods, allocation concealment, extent of blinding (participants, data collectors, outcome assessors, and data analysis), incomplete outcome data, selective reporting, and other biases. The evaluations were scaled as a low, unclear, and high risk of bias, according to the criteria for judging the risk of bias provided by the Cochrane handbook [35]. 

### 2.7. Statistical Analysis

Statistical analysis was carried out using Statistica 13.0 software (StatSoft, Tulsa, OK, USA), and the therapeutic effect of vitamin D on the PCOS patients was estimated by the standardized mean difference (SMD) with 95% confidence intervals (CI). The overall SMD was evaluated by the Z-test. Heterogeneity across studies was evaluated using Cochran’s *Q*-statistic (with *p* < 0.1 implying a significant difference) and the *I*^2^-statistic (with *I*^2^ = 0% meaning no heterogeneity and *I*^2^ = 100% meaning maximal heterogeneity). A random-effect model was employed when *p* < 0.1 and *I*^2^ > 70%; *p* > 0.1 meant there was no heterogeneity among the studies, so a fixed-effect model was applied. Publication bias was assessed by a funnel plot. All statistical tests were two-sided, and *p* values below 0.05 were considered statistically different.

## 3. Results

### 3.1. Search Results

**Figure 1:**
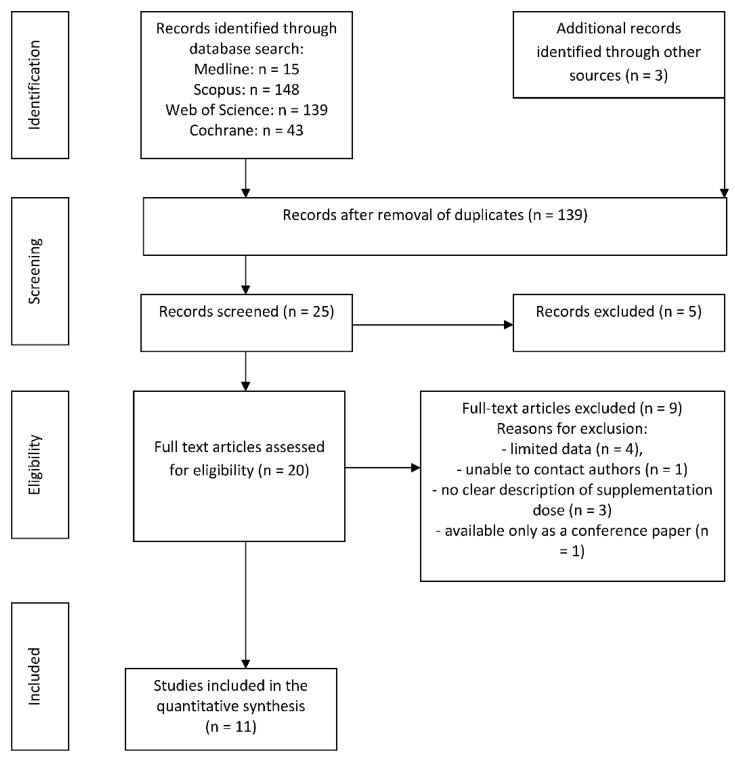
The search process.

A flow chart of the extraction is presented in Figure 1. Through out the initial search strategy, we identified 348 articles; following analysis of their titles and abstracts section, twenty publications were selected for full-text review. Finally, after the removal of duplicates and publications with insufficient data, 11 RCTs met the inclusion criteria and were included in the final analysis.

### 3.2. Population and Study Characteristics

Baseline characteristics, number of study participants, study duration, type of intervention, and weight-loss outcomes are presented in Table 1, Table 2 and Table 3, respectively. Overall, 601 patients were included from the eleven selected studies. Although the date of publication of the selected papers was not limited, all the articles included in the systematic review were published after 2012. The number of participants in the studies ranged from 28 to 90 [20,22]. The age of the women ranged from 18 to 40 and the BMI value ranged from 24.2 [19] to 37.2 kg/m^2^ [20]. Patients were of the following ethnicities: Asian (89%) [16,17,18,19,21,22,23,24], African-America (0.5%) [15], and Hispanic (6%) [15]. The study of Raja-Khan et al. [20] lacked information on the ethnicity of the study population. Studies were designed as RCTs. In interventions employing supplementation with cholecalciferol, the dose ranged from 200 IU/d [24] to 60,000 IU/weekly [21], with 2000 IU/d (0.5 μd/d) calcitriol [24] and 200 IU/d as a co-supplementation [2,19].

### 3.3. Effects of Vitamin D Supplementation on 25(OH)D Levels

**Figure 2:**
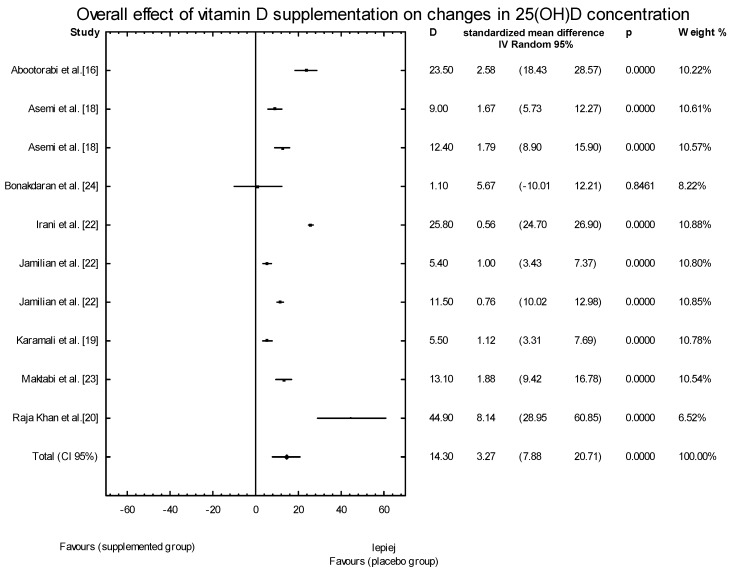
Overall effect of vitamin D supplementation on changes in 25(OH)D concentration in PCOS patients. Heterogeneity: *Q* = 902.5137, T^2^ = 163.1096, df = 9 (*p* < 0.0001); *I*^2^ = 99.0%.

In the eleven studies, we assessed the effects of vitamin D supplementation on 25(OH)D serum concentrations. The baseline serum concentrations of 25(OH)D in the supplemented groups (SG) ranged from 6.9 ± 4.3 ng/mL [17] to 19.9 ± 9.5 ng/mL [20], and were similar to the values observed in the placebo groups. The low concentrations of 25(OH)D suggest the widespread presence of vitamin D deficiency or insufficiency in these groups of women with PCOS. In ten of the eleven studies, we observed an increase in the serum concentrations of 25(OH)D following supplementation with vitamin D, leading to improved serum levels ranging from 18.5 ± 4.9 ng/mL [22] to 67.4 ± 28.6 ng/mL [20] (Table 2). When the meta-analysis was conducted, significant overall effects of vitamin D supplementation on 25(OH)D concentrations were seen (SMD: 4.16, 95% CI: 3.92, 20.23, *p* = 0.0037; Figure 2).

### 3.4. Effects of Vitamin D Supplementation on Changes in Parameters Related to Insulin Resistance

Changes in fasting plasma glucose concentrations after vitamin D supplementation were analyzed in the nine studies determined to be eligible during the search process [11,12,13,14,15,16,17,18,19,20].

The average baseline blood glucose concentration ranged from 81.6 ± 10.00 mg/dL [14] to 99.8 ± 10.1 mg/dL [13] in the SG. Similar results were observed in the placebo groups. Following the intervention, mean fasting glucose concentrations decreased (by 6.3%) in individuals who received vitamin D supplementation alone (three selected studies) or as a co-supplement (two selected studies), while seven studies showed no differences in fasting blood glucose levels (Table 3).

**Figure 3:**
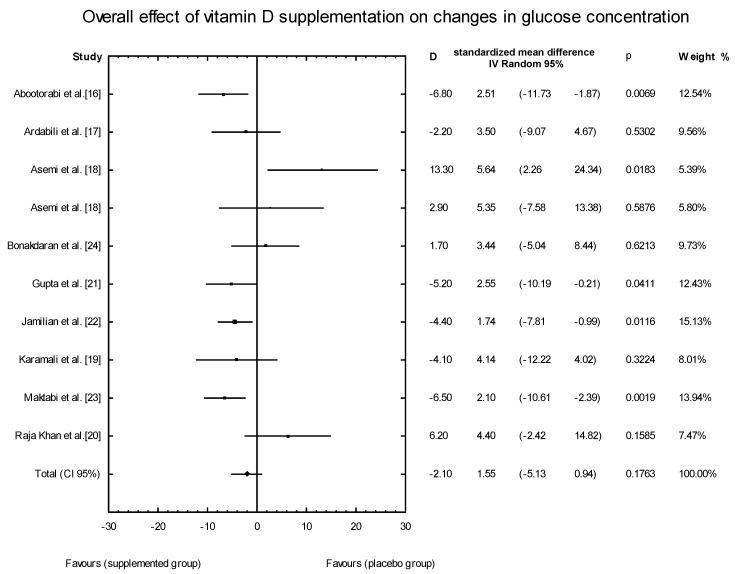
Overall effect of vitamin D supplementation on changes in glucose concentration in PCOS patients. Heterogeneity: *Q* = 21.6993, T^2^ = 12.4443, df = 9 (*p* = 0.0099); *I*^2^ = 58.52%.

**Figure 4:**
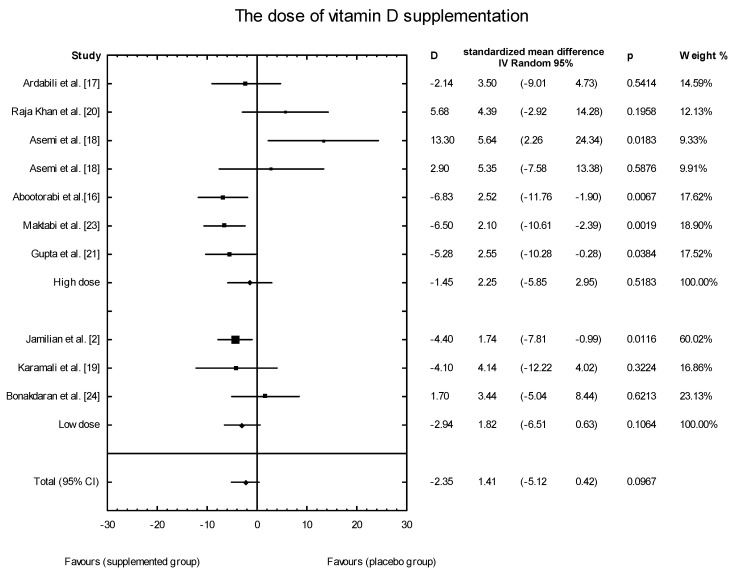
The effect of different doses of vitamin D supplementation on glucose concentration in PCOS patients. High dose D = −1.45, *p* = 0.5183, T^2^ = 22.30; Low dose = D = −2.94, *p* = 0.1064; T^2^ = 2.49, Test for overall effects: Z = 0.4933 (*p* = 0.6218).

**Figure 5:**
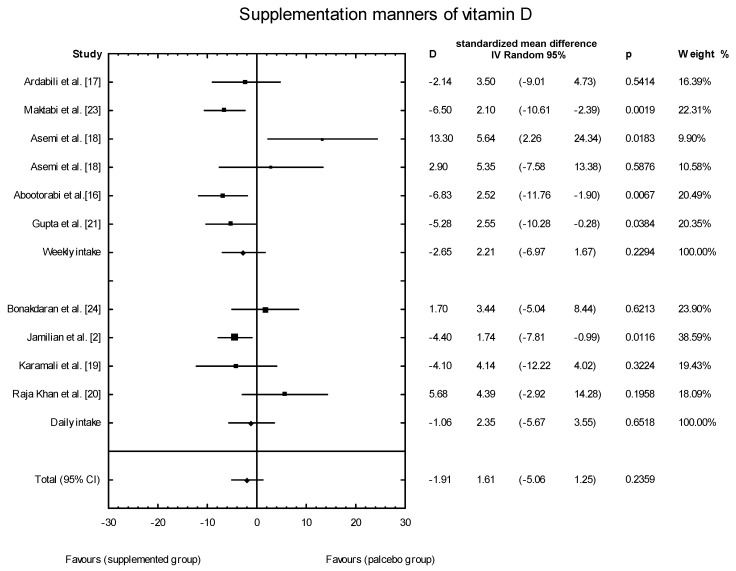
The effect of supplementation manners on glucose concentration in PCOS patients. Daily intake D = −1.06, *p* = 0.6518, T^2^ = 11.29; Weekly intake D = −2.65, *p* = 0.229; T^2^ = 17.24, Test for overall effects: Z = 0.4933 (*p* = 0.6218).

**Figure 6:**
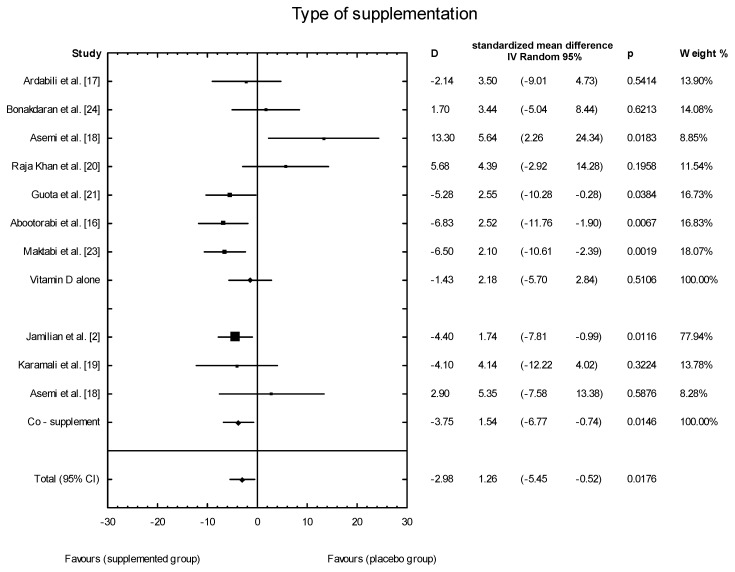
The effect of different types of supplementation on glucose concentration in PCOS patients. Vitamin D alone: D = −1.43, *p* = 0.5106, T^2^ = 21.86, Co-supplementation: D = −1.43, *p* = 0.0146, T^2^ = 0, Test for overall effects: Z = 0.8704 (*p* = 0.3841).

The meta-analysis showed no significant overall effect of vitamin D supplementation on fasting glucose concentrations (SMD: 1.54, 95% CI: −5.17, 0.85, *p* = 0.16, Figure 3), or when different doses of vitamin D were considered (high dose: SMD: 2.25; 95% CI: −5.85, 2.95; *p* = 0.52; low dose: SMD: 1.82; 95% CI: −6.51, 0.63; *p* = 0.11) (Figure 4), or when supplementation manner was considered (weekly dose: SMD:2.21; 95% CI: −6.97, 1.67; *p* = 0.23; daily dose: SMD: 2.35; 95% CI: −5.67, 3.55; *p* = 0.65) (Figure 5). However, the type of supplementation proved significant (vitamin D alone: SMD: 2.18; 95% CI: −5.70, 0.28; *p* = 0.51; co-supplement: SMD: 1.54; 95% CI: −6.77, −0.74; *p* = 0.0146) (Figure 6).

The effect of vitamin D supplementation on fasting insulin secretion was evaluated in nine studies. In the SG, whether alone or as a co-supplement, the mean fasting plasma insulin concentrations ranged from 9.60 ± 4.5 μLU/L [23] to 26.31 ± 9.60 μLU/L [20]. After the intervention, the mean fasting insulin levels had decreased significantly (by 22%) in individuals who received vitamin D supplementation alone (two studies) and as a co-supplement (two studies), while seven of the studies showed no differences in fasting blood insulin levels (Table 3).

**Figure 7:**
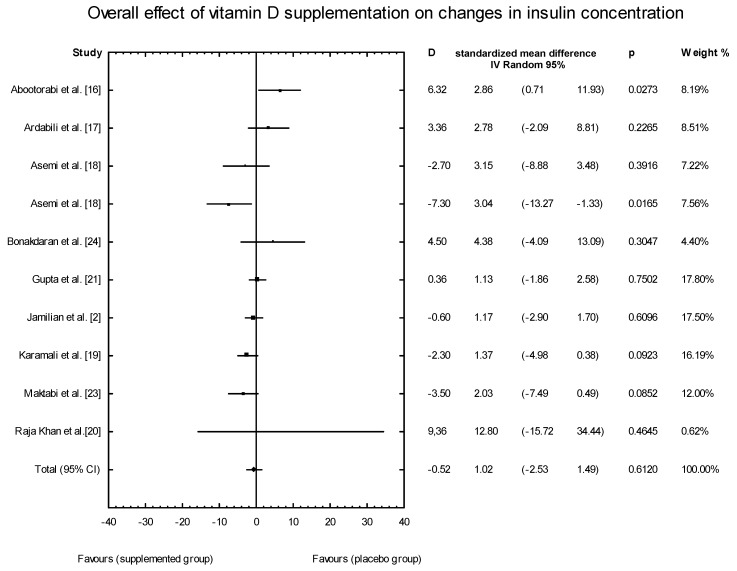
Overall effect of vitamin D supplementation on changes in insulin concentration in PCOS patients. Heterogeneity: Q = 19.4134; T^2^ = 4.6214, df = 9 (*p* = 0.0219); *I*^2^ = 53.64%.

**Figure 8:**
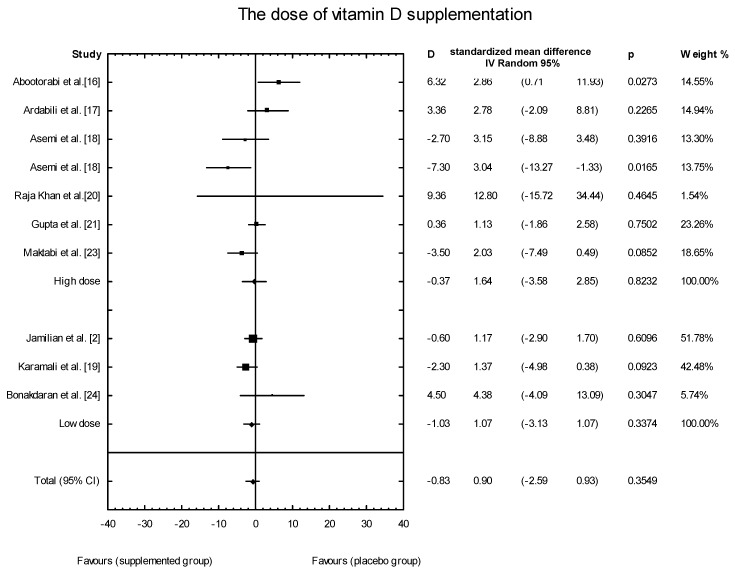
Effect of different doses of vitamin D supplementation on insulin concentration in PCOS patients, high dose D = −0.37, *p* = 0.8232, T^2^ = 10.28, low dose D = −1.03, *p* = 0.3374, T^2^ = 0.84. Test for overall effects: Z = 33.84 (*p* = 0.7351).

**Figure 9:**
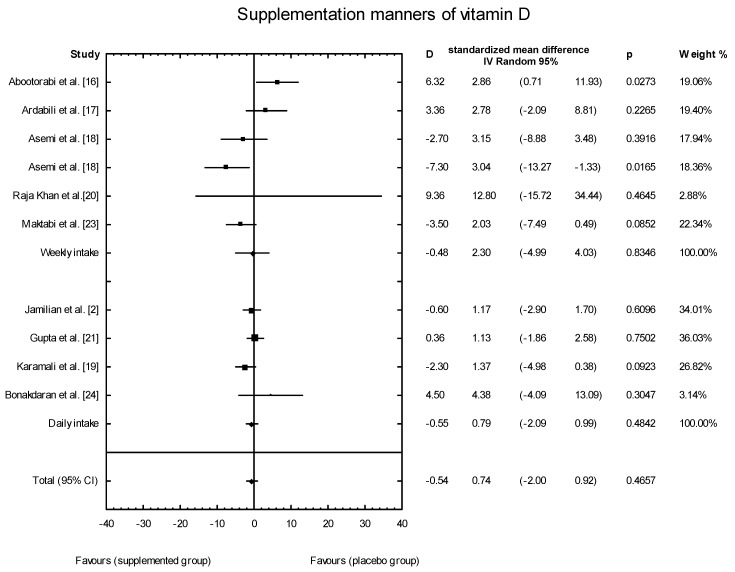
Effect of different supplementation manners on insulin concentration of vitamin D in PCOS patients. Daily in take D = −0.55, *p* = 0.4842, T^2^ = 0.44; weekly in take D = −0.48, *p* = 0.8346, T^2^ = 19.51; Test for overall effects: Z = 0.0287 (*p* = 0.9771).

**Figure 10:**
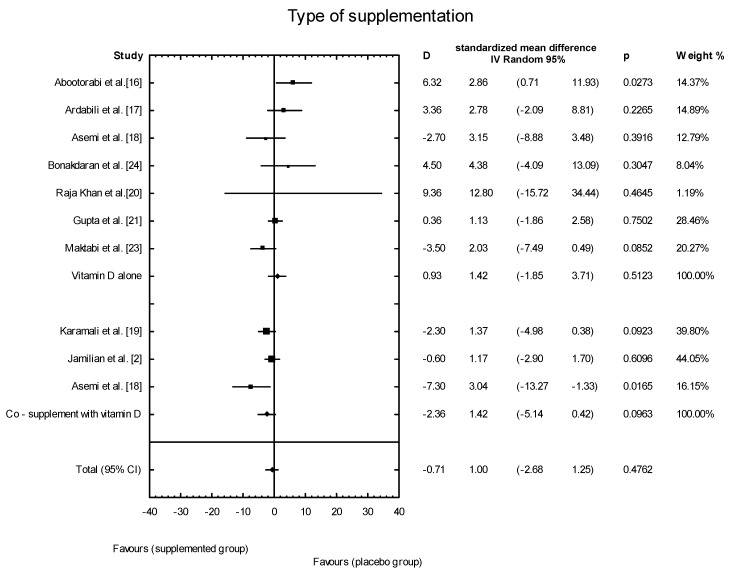
Effect of different types of supplementation on insulin concentration in PCOS patients. Vitamin D alone: D = 0.93, *p* = 0.5123, T^2^ = 3.19; co-supplement: D = −0.93, *p* = 0.5123, T^2^ = 5.78; Test for overall effects: Z = 1.6394 (*p* = 0.1011).

The meta-analysis showed no significant overall effect of vitamin D supplementation on insulin levels (SMD: 1.02, 95% CI: −2.53, 1.49, *p* = 0.6120, Figure 7), and no such effect when the different doses of vitamin D were taken into consideration (high dose: SMD:1.64; 95% CI: −3.58, 2.85; *p* = 0.82; low dose: SMD: 0.90; 95% CI: −2.59, 0.93; *p* = 0.35) (Figure 8), when supplementation manners were considered (weekly intake: SMD: −2.30; 95% CI: −4.99, 4.03; *p* = 0.83;daily intake: SMD: 0.79; 95% CI: −2.08, 0.99; *p* = 0.48) (Figure 9), or when the type of supplementation was considered (vitamin D alone: SMD: 1.42; 95% CI: −1.85, 3.71; *p* = 0.51; co-supplement: SMD: 1.42; 95% CI: −5.14, 0.42; *p* = 0.09) (Figure 10).

**Figure 11:**
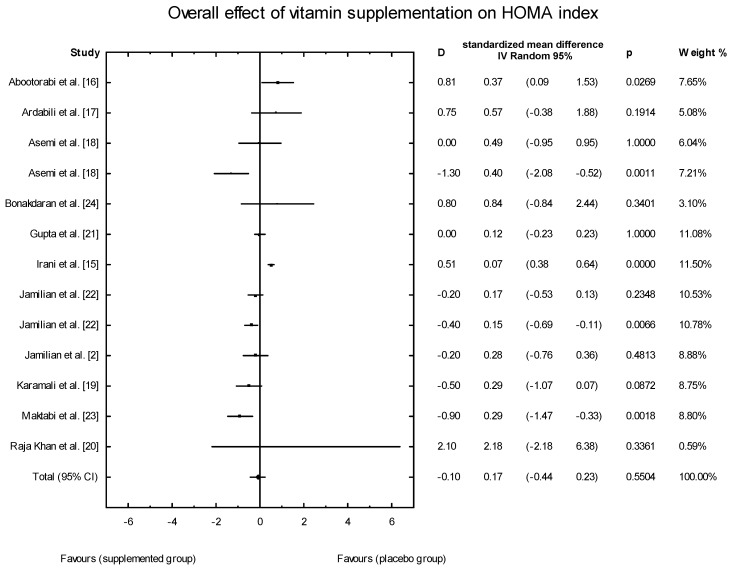
Overall effect of vitamin D supplementation on the HOMA-IR index in PCOS patients. Heterogeneity: Q = 86.9395; T^2^ = 0.2529, df = 12 (*p* = 0.000); *I*^2^ = 86.20%.

**Figure 12:**
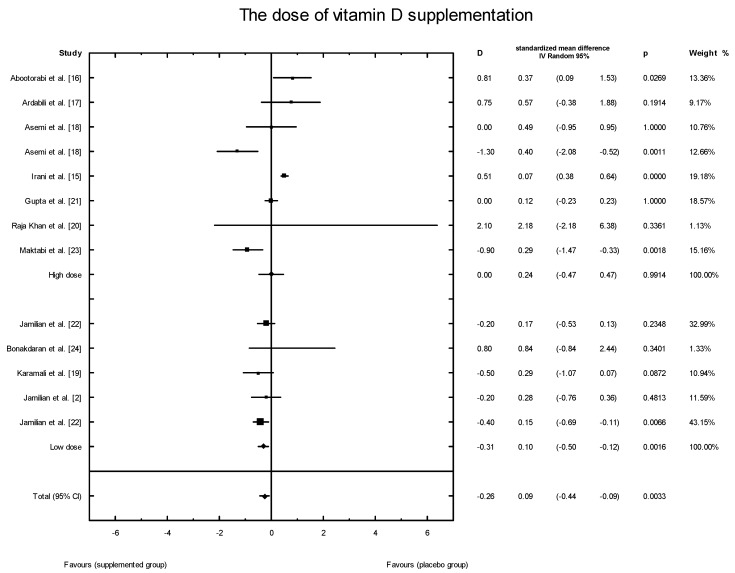
Effect of different doses of vitamin D supplementation on HOMA-IR index in PCOS patients. High dose D = 0.18, *p* = 0.9914, T^2^ = 0.29; Low dose D = −0.31, *p* = 0.0016, T^2^ = 0.00; Test for overall effects: Z = 1.1770 (*p* = 0.2392).

**Figure 13:**
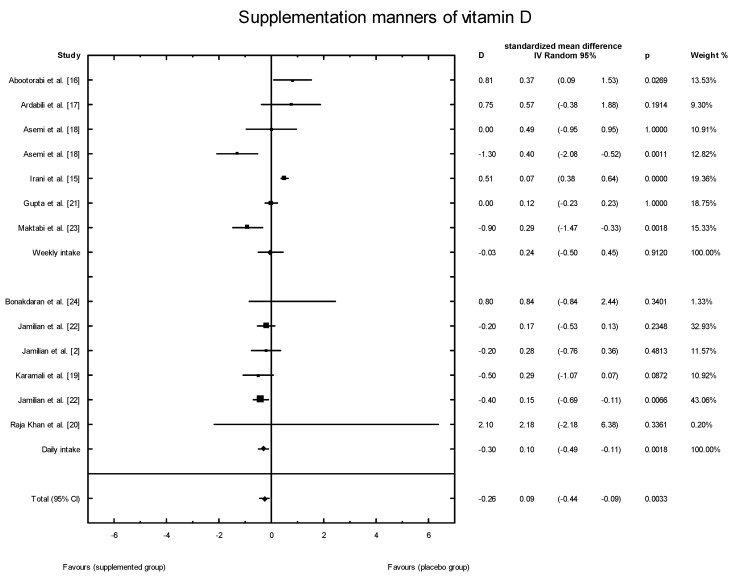
Effect of different manners of supplementation with vitamin D on HOMA-IR index in PCOS patients. Weekly in take D = −0.03, *p* = 0.9120, T^2^ = 0.30; Daily in take D = −0.30, *p* = 0.0018, T^2^ = 0.00; Test for overall effects: Z = 1.0570 (*p* = 0.2905).

**Figure 14:**
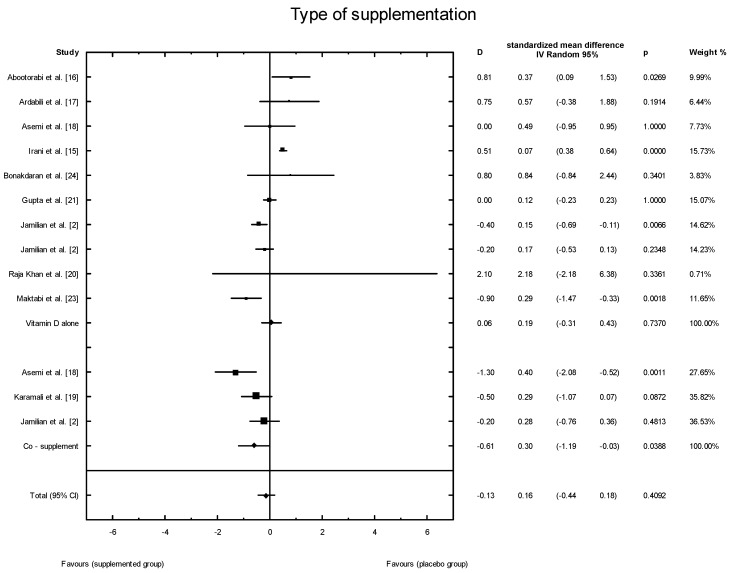
Effect of different types of vitamin D supplementation on HOMA-IR index in PCOS patients. Vitamin D alone: D = 0.06, *p* = 0.7370, T^2^ = 0.22; Co-supplement: D = −0.61, *p* = 0.0388, T^2^ = 0.16; Test for overall effects: Z = 1.9241 (*p* = 0.0543).

The effects of vitamin D supplementation on the HOMA-IR index were analyzed in all the selected studies. In the SG, the mean HOMA-IR ranged from 2.07 ± 0.37 to 5.47 ± 1.82 (Table 3). In five of the analyzed studies, the values of the HOMA-IR index decreased after vitamin D supplementation, but there were no significant effects of vitamin D supplementation on the HOMA-IR index overall (SMD: 0.17; 95% CI: −0.44, 0.23; *p* = 0.55, Figure 11). However, when the meta-analysis was conducted in specific subgroups, it was found that the dosage, supplementation manner, and type of vitamin D had a significant effect on HOMA-IR value (high dose of vitamin D: SMD: 0.24; 95% CI: −0.47, 0.47; *p* = 0.99; low dose: SMD: 0.10; 95% CI: −0.50, −0.12; *p* = 0.001; Figure 12;weekly intake: SMD: 0.24; 95% CI: −0.50, 0.45; *p* = 0.91; Figure 13; daily intake: SMD: 0.10; 95% CI: −0.49, −0.11; *p* = 0.002). Furthermore, the type of vitamin D supplement was also an important factor (vitamin D alone: SMD: 0.19; 95% CI: −0.31, 0.43; *p* = 0.74; co-supplement: SMD: 0.30; 95% CI: −1.19, −0.03; *p* = 0.04) (Figure 14).

### 3.5. Publication Bias

**Figure 15:**
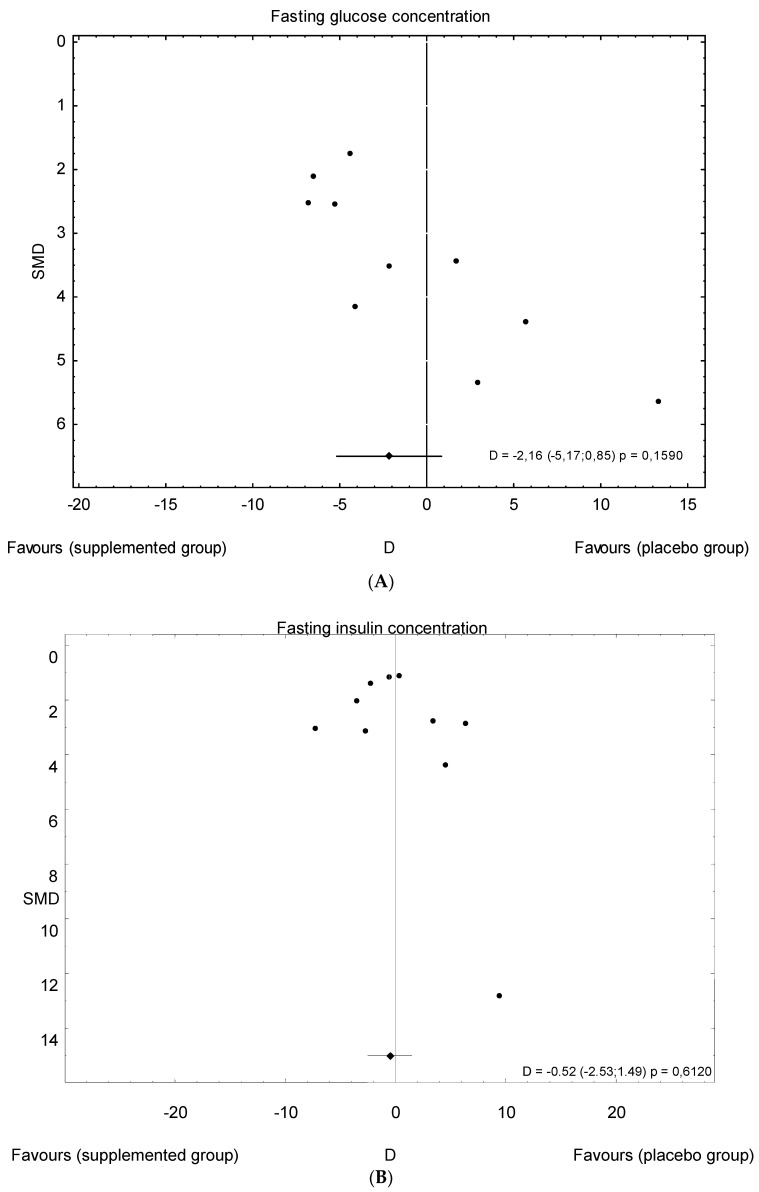

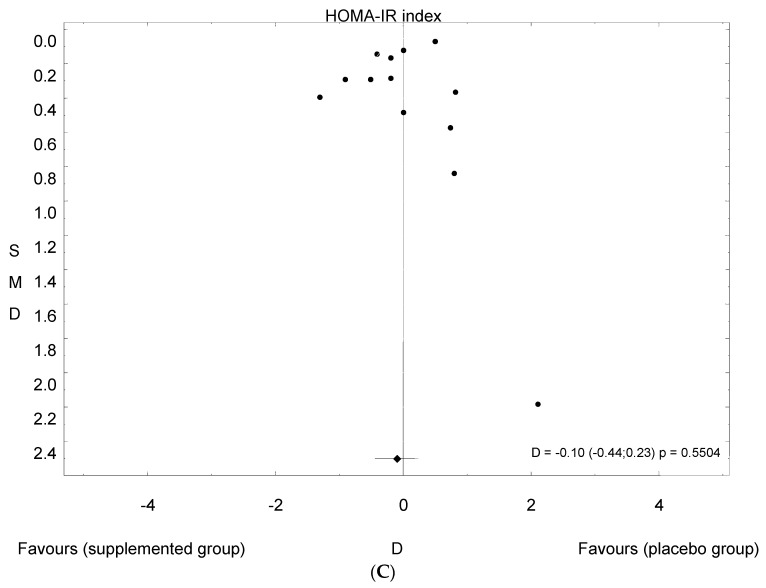
Funnel plot of standard error by standard differences in the means of plasma: (**A**) fasting glucose concentrations; (**B**) fasting insulin concentrations; and (**C**) HOMA-IR index in selected randomized controlled trials.

The funnel plots for serum vitamin D and fasting glucose, fasting insulin concentrations, and HOMA-IR indicate that there is a lack of smaller studies showing negative effects of vitamin D supplementation on vitamin D levels and positive effects on fasting glucose, fasting insulin concentrations, and HOMA-IR; this suggests that there is a possible, though minimal, publication bias. However, the number of studies available for each meta-analysis was no more than seven, which may be too small to assess the publication bias through funnel plots (Figure 15A–C).

## 4. Discussion

We have presented here the effect of vitamin D supplementation on insulin resistance in women with PCOS. Our results indicate that vitamin D supplementation, in combination with the supplementation of calcium, vitamin K, zinc, and magnesium, may help improve the metabolic profile of women diagnosed with PCOS in terms of glucose concentration and HOMA-IR. Lower daily doses (<4000 IU/d) of vitamin D also helped lower the HOMA index.

Vitamin D deficiency is a common health problem that may affect up to half of the general adult population [36]. A relatively higher prevalence of vitamin D deficiency is observed among women with PCOS: approximately 67–85% of women with PCOS have insufficient levels of vitamin D [36]. In our meta-analysis, all patients had insufficient 25(OH)D levels. However, according to Sempos et al. [32], most but not all women had insufficient 25(OH)D levels (only in Raja-Khan et al. (2014)did women with PCOS receiving placebo have a mean of 25(OH)D concentrations above 20 ng/mL). Sempos et al. [32] indicate that, if this controversy over the definition of hypovitaminosis D is to be resolved, it will be necessary: (i) to standardize the measurement of serum total 25(OH)D in vitamin D research, as well as harmonize the measurement of other possible markers of vitamin D status; and (ii) to develop a rickets registry that includes a precise case definition of nutritional rickets, including other risk factors for nutritional rickets, and standardized measurements of 25(OH)D and vitamin D metabolites. Yet, even taking this into account, there are rational premises for supplementing women with PCOS with vitamin D.

Data from the collected trials with 601 women with PCOS show that vitamin D supplementation significantly increased 25(OH)D levels compared to the placebo group, with a mean difference of 17.27 ng/mL (95% CI, D = 12.07; Figure 1). This response was highly heterogeneous (*Q* = 902.5137, *I*^2^ = 99.0% and T^2^ test for heterogeneity 103.1096).

Many authors have suggested that there is an association between vitamin D status and metabolic dysfunctions—particularly insulin resistance—in women with PCOS. However, the results of randomized controlled trials that assessed the effect of vitamin D treatment of PCOS patients on glucose homeostasis remain inconclusive [15,18,24,37]. In a previous meta-analysis that only included the results of controlled randomized trials, the fasting glucose, fasting insulin, serum HOMA-IR, and QUICKI of PCOS patients did not change after supplementation with vitamin D [38,39]. The authors of that analysis suggested that several factors might explain these contrary results, including the different follow-up period of vitamin D supplementation, or supplementation with vitamin D alone or with other micronutrients. Similarly, in our meta-analysis, when the vitamin D supplementation was considered in the overall view, the regime did not significantly affect fasting glucose, fasting insulin, or HOMA-IR in women with PCOS. We thus decided to conduct a much more detailed analysis. It was recognized that the dose of vitamin D (low or high), the frequency of supplementation (daily or weekly), and the form in which vitamin D was given (alone or as a co-supplement) all held a crucial significance for glucose homeostasis. It was also seen that HOMA-IR decreased for low doses of vitamin D (≤4000 IU/d).This could be the result of the more regular absorption of vitamin D_3_ in the gut or better compliance.

Moreover, vitamin D in combination with other nutrients (especially calcium) significantly affected fasting glucose levels and HOMA-IR. There is still some conjecture as to whether calcium and vitamin D, alone or in combination, can affect insulin-secreting cells. It is known that insulin secretion is a calcium-dependent process; thus, vitamin D may influence pancreatic β-cells via the regulation of calcium concentrations [40]. Also, a beneficial effect of combined vitamin D and calcium on markers of glucose metabolism and lipid profiles in patients with type 2 diabetes mellitus [41] has previously been reported, but data on the effect of co-supplementation with vitamin D, vitamin K, magnesium, and zinc on markers of insulin metabolism and lipid profiles among vitamin D-deficient subjects with PCOS are still scarce.

It should be highlighted that the Endocrine Society Guidelines [31] recommend supplementation with 50,000 IU vitamin D_3_ once per week for eight weeks (this was the minimum time of supplementation in our meta-analysis).The results of the meta-analysis of Xue et al. [34] show that supplementation with a low dose of vitamin D was enough to reduce triglyceride levels, while supplementation with high doses was not beneficial. Sanders et al. [42] pointed out that high doses of vitamin D supplementation were harmful to health, because bolus doses of more than 500,000 IU of vitamin D_3_might increase the risk of fracture, alter biochemical markers, and caused issues with tolerability, such as gastrointestinal upset. However, in the studies included in our meta-analysis, no side effects of high doses of vitamin D supplementation were observed and weekly high doses also proved ineffective in lowering HOMA-IR. 

In seeking to justify the combination of vitamin D with other ingredients and the use of co-supplementation in the treatment of insulin resistance in women with PCOS, it should be noted that magnesium participates in phosphorylation reactions of the signaling pathway of insulin; it is also part of the Mg^2+^-ATP complex. In addition, zinc intake may modulate the protein tyrosine phosphatase 1B, a key regulator of the phosphorylation state of the insulin receptor. Zinc has been documented to inhibit 5-α-reductase, which catalyzes the transformation of testosterone to its nonaromatizable form, dihydrotestosterone (DHT). Thus, elevated levels of zinc may help reduce PCOS-associated hyperandrogenemiaby inhibiting the transformation of testosterone to its active form, DHT. Furthermore, both animal and human studies have suggested that the adipokine regulatory ability, anti-inflammatory properties, and lipid-lowering effects of the vitamin K-dependent protein (osteocalcin) may mediate the beneficial functions of vitamin K in insulin sensitivity and glucose tolerance. It has also been reported that supplementation with vitamin D, vitamin K, and calcium may improve metabolic status through their effects on the up-regulation of the insulin receptor genes, the regulation of insulin secretion from pancreatic β-cells, and the enhancement of β-cell proliferation and adiponectin expression [18,38,39].

The strengths of this meta-analysis include its comprehensive literature search, the specified inclusion and exclusion criteria for the studies, the explicit methods of data extraction, the measures taken to reduce the influence of bias, and the assessment of heterogeneity. Our work also has some limitations. Firstly, it is possible that some studies published in the grey literature were omitted in the literature search. Secondly, there were many variations between the studies, including the nationality and race of the patients, age, methodology, drug doses, duration of treatment, and follow-up, as well as vitamin D formulation. Grossmann and Tangpricha identified four clinical studies that compared two different vehicles for delivering vitamin Din their systematic review. In healthy subjects, vitamin D in an oil vehicle produced a greater 25(OH)D response than vitamin D in a powder or an ethanol vehicle [43]. On the other hand, Coelho et al. indicated that vitamin D_3_ entering the bloodstream is not affected by capsule formulation [44]. Since there have been limited studies that have compared the effect of the vehicle substance on vitamin D bioavailability, future studies should examine bioavailability across different vehicle substances, such as oil, lactose powder, and ethanol [43]. Moreover, most studies included in the meta-analysis were conducted in Iran, where women’s skin is largely covered with clothes, thus preventing the sun stimulating vitamin D production; this may be associated with vitamin D deficiency. It may thus be incorrect to assume that the results of these Iranian studies can be directly compared with those of studies on Caucasian women.

However, in general, there have been very few works dealing with the effect of co-supplementation on the parameters of insulin resistance in PCOS women. Our results thus need to be further validated by larger prospective studies.

## 5. Conclusions

In conclusion, this systematic review provides evidence that vitamin D co-supplementation among PCOS women is effective in decreased fasting glucose concentration and HOMA-IR treatment. Additionally, HOMA-IR also decreased when vitamin D alone was taken every day (not once a week) and in low doses (<4000 IU/d).

## Figures and Tables

**Table 1:** Characteristics of the included studies and study populations (*n* = 601).

Study	Country	Subjects (*n*)	Intervention	Supplemented Dose of Vitamin D	Vitamin D Supplements: Formulation and Manufacturer	Time of Intervention	Ethnicity
Abootorabi et al. [16], 2017	Iran	36	Cholecalciferol (*n* = 19) Placebo (*n* = 17)	50,000 IU/weekly	Capsules: D-Vitamin 50,000; Zahravi Pharm Co, Tabriz, Iran	8 weeks	Asian: 100%
Ardabili et al. [17], 2012	Iran	50	Cholecalciferol (*n* = 24) Placebo (*n* = 26)	50,000 IU every 20 days Placebo	Capsules: D-Vitin 50,000, Zahravi Pharm Co, Tabriz, Iran	60 weeks	Asian: 100%
Asemi et al. [18], 2014 ^a^	Iran	78	Cholecalciferol (*n* = 26) Cholecalciferol + Calcium(*n* = 26) placebo (*n* = 26)	50,000 IU/week 50,000 IU/week + 1000 mg/d Placebo	Tablets: Dana Pharmaceutical Company (Tabriz, Iran) and Barij Essence Pharmaceutical Company (Kashan, Iran).	8 weeks	Asian: 100%
Bonakdaran et al. [24], 2012 ^b^	Iran	31	Calcitriol (*n* = 15) Placebo (*n* = 16)	200 IU/d Placebo	Capsules: Zahravi, Tabriz, Iran	12 weeks	Asian: 100%
Gupta et al. [21], 2017	India	50	Cholecalciferol (*n* = 25) Placebo (*n* = 25)	60,000 IU/weekly Placebo	Not given	12 weeks	Asian: 100%
Irani et al. [15], 2015	USA	53	Cholecalciferol (*n* = 35) Placebo (*n* = 18)	50,000 IU/weekly Placebo	Capsules: manufacturer not given	8 weeks	Vitamin D group: Hispanic: 69.4%; Asian: 25%; Black: 5.5% Placebo group: Hispanic: 72.2%; Asian: 22.2%; Black: 5.5%
Jamilian et al. [2], 2017	Iran	90	Cholecalciferol (*n* = 30) Cholecalciferol (*n* = 30) Placebo (*n* = 30)	1000 IU/d 4000 IU/d Placebo	Capsules: Zahravi Pharmaceutical Company (Tabriz, Iran) and Barij Essence Pharmaceutical Company Kashan, Iran	12 weeks	Asian: 100%
Jamilian et al. [22], 2017	Iran	60	Cholecalciferol+Magnesium+Zinc+Calcium (*n* = 30) Placebo (*n* = 30)	200 IU + 100 mg + 4 mg + 400 mg/d Placebo	Tablets: Vitane (Wolfratshausen, Germany) and Barij Essence Pharmaceuticals (Kashan, Iran),	12 weeks	Asian: 100%
Karamali et al. [19], 2018	Iran	55	Cholecalciferol + Calcium + Vitamin K (*n* = 28) Placebo (*n* = 27)	200 IU + 500 mg + 90 μg/d Placebo	Capsules: Arian SalamtSina (Tehran, Iran) and Barij Essence Pharmaceutical Company(Kashan, Iran)	8 weeks	Asian: 100%
Maktabi et al. [23], 2017	Iran	70	Cholecalciferol (*n* = 35) Placebo (*n* = 35)	50,000 IU every 2 weeks Placebo	Capsules: Zahravi, Tabriz, Iran	12 weeks	Asian: 100%
Raja-Khan et al. [20], 2014	USA	28	Cholecalciferol (*n* = 13) Placebo (*n* = 15)	12,000 IU/d Placebo	Gel caps: Maximum D3 (with soy lecithin oil): BTR Group, Inc. (Pittsfield, IL, USA)	12 weeks	N/A

N/A: Not available. ^a^ In this study, additional supplementation with calcium alone was also tested. These results were not taken into account in this meta-analysis. ^b^ In this study, asecond control grouponly took metformin. These results were not taken into account in this meta-analysis.

**Table 2:** Mean changes in 25(OH)D (ng/mL) concentration after supplementation with vitamin D in the supplemented and placebo groups of the selected studies.

Study	Supplemented Dose of Vitamin D	Study Groups	Age (Years) Mean ± SD	BMI (kg/m^2^) Mean ± SD	Serum 25(OH)D (ng/mL)Concentration Mean ± SD	
					Before Supplementation	After Supplementation
Abootorabi et al. [16], 2017	Cholecalciferol Placebo	SG PG	26.2 ± 4.6 22.8 ± 4.4	N/A	8.65 ± 4.3 9.80 ± 5.1	36.9 ± 8.4 *** 13.4 ± 7.1
Ardabili et al. [17], 2012	Cholecalciferol Placebo	SG PG	26.8 ± 4.7 27.0 ± 3.7	29.1 ± 4.6 28.3 ± 3.5	6.9 ± 2.8	23.4 ± 6.1 ***
Asemi et al. [18], 2014^a^	Cholecalciferol Cholecalciferol + Calcium placebo	SG SG PG	25.6 ± 4.4 24.9 ± 5.1 24.3 ± 5.2	29.3 ± 3.9 27.3 ± 5.3 27.5 ± 5.2	11.6 ± 4.7 15.1 ± 3.6 14.0 ± 4.1	23.4 ± 7.1 ** 26.8 ± 7.8 *** 14.4 ± 4.7
Bonakdaran et al. [24], 2012^b^	Calcitriol Placebo	SG PG	24.7 ± 3.3 25.2 ± 7.9	24.8 ± 5.3 25.3 ± 5.1	11.4 ± 8.2 19.9 ± 16.5	20.1 ± 16.2 19.0 ± 15.3
Gupta et al. [21], 2017	Cholecalciferol Placebo	SG PG	26.0 ± 3.7 26.6 ± 3.7	24.9 ± 2.8 25.6 ± 2.0	18.56 ± 9.7	44.90 ± 9.04 ***
Irani et al. [15], 2015	Cholecalciferol Placebo	SG PG	30.5 ± 1.0 29.6 ± 1.7	30.0 ± 1.0 28.0 ± 1.6	16.3 ± 0.9 17.0 ± 1.8	43.2 ± 2.4 ** 17.4 ± 1.9
Jamilian et al. [22], 2017	Cholecalciferol+Magnesium+Zinc+Calcium Placebo	SG PG	18–40	N/A	+7.9 ± 8.4 *** ^b^ +0.1 ± 8.4	
Jamilian et al. [2], 2017	Cholecalciferol Cholecalciferol Placebo	SG low dose SG high dose PG	26 ± 5.0 28 ± 5.0 25 ± 5.0	33 ± 5 31 ± 6 30 ± 6	12.6 ± 3.4 12.6 ± 2.7 12.9 ± 2.4	18.5 ± 4.9 * 24.6 ± 3.3 * 13.1 ± 2.5
Karamali et al. [19], 2018	Cholecalciferol + calcium + vitamin K Placebo	SG PG	23.5 ± 4.2 23.3 ± 3.4	24.2 ± 4.8 24.3 ± 3.9	14.7 ± 2.5 14.8 ± 3.9	20.0 ± 3.0 *** 14.5 ± 5.0
Maktabi et al. [23], 2017	Cholecalciferol Placebo	SG PG	18–40 ^a^	N/A	12.8 ± 4.5 14.5 ± 5.1	27.5 ± 9.8 *** 14.4 ± 5.2
Raja-Khan et al. [20], 2014	Cholecalciferol Placebo	SG PG	28.2 ± 5.2 28.7 ± 5.6	37.2 ± 4.5 35.1 ± 9.8	19.95 ± 9.5 22.20 ± 6.9	67.4 ± 28.6 *** 22.5 ± 7.0

N/A: not available. SG: supplemented group. PG: placebo group. ^a^ In this study, additional supplementation with calcium alone was also tested. These results were not taken into account in this meta-analysis. ^b^ In this study, a second control group only took metformin, * *p* < 0.05; ** *p* < 0.01; *** *p* < 0.001.

**Table 3:** Mean changes in fasting glucose concentration (mg/dL), fasting insulin level (μLU/L), and value of HOMA-IR index before and after supplementation with vitamin D in the supplemented and placebo groups in the selected studies.

Study	Supplemented Dose of Vitamin D	Study Groups	Fasting Glucose (mg/dL) Mean ± SD		Fasting Insulin (μLU/mL) Mean ± SD		HOMA-IR Mean ± SD	
			Before	After	Before	After	Before	After
Abootorabi et al. [16], 2017	Cholecalciferol Placebo	SG PG	86.7 ± 6.8 84.2 ± 5.9	79.1 ± 7.1 *** 85.9 ± 7.9	14.7 ± 7.5 8.2 ± 5.8	15.9 ± 7.3 9.6 ± 3.3	2.8 ± 1.3 1.7 ± 1.2	2.8 ± 1.4 2.0 ± 0.7
Ardabili et al. [17], 2012	Cholecalciferol Placebo	SG PG	99.8 ± 10.1 101.5 ± 10.6	96.6 ± 9.9 98.8 ± 14.6	12.5 ± 15.1 9.9 ± 5.3	13.3 ± 9.7 10.0 ± 4.1	3.2 ± 4.1 2.5 ± 1.4	3.2 ± 2.6 2.5 ± 1.1
Asemi et al. [18], 2014 ^a^	Cholecalciferol Cholecalciferol + calcium placebo	SG SG PG	87.3 ± 16.4 81.6 ± 10.0 67.6 ± 11.7	86.8 ± 16.1 76.4 ± 13.3 * 73.5 ± 23.8	13.5 ± 9.9 11.1 ± 14.2 12.0 ± 5.6	12.4 ± 5.5 * 7.8 ± 3.6 * 15.1 ± 7.1	3.1 ± 2.8 2.2 ± 2.8 2.0 ± 1.1	2.8 ± 1.6 * 1.5 ± 0.7 * 2.8 ± 1.9
Bonakdaran et al. [24], 2012 ^b^	Calcitriol Placebo	SG PG	81.7 ± 8.6 86.3 ± 5.4	89.0 ± 12.3 87.3 ± 5.3	18.3 ± 30.4 13.6 ± 14.6	13.1 ± 14.8 8.6 ± 5.0	4.2 ± 6.8 2.8 ± 2.9	2.7 ± 3.1 1.9 ± 1.0
Gupta et al. [21], 2017	Cholecalciferol Placebo	SG PG	88.2 ± 9.3 91.3 ± 8.4	82.4 ± 8.0 * 87.6 ± 9.9	10.3 ± 20.0 4.6 ± 0.6	5.0 ± 3.2 * 4.6 ± 0.6	2.4 ± 4.9 1.0 ± 0.6	1.0 ± 0.6 * 1.0 ± 0.2
Irani et al. [15], 2015	Cholecalciferol Placebo	SG PG	N/A	N/A	N/A	N/A	2.1 ± 0.4 1.6 ± 0.3	2.0 ± 0.2 1.5 ± 0.2
Jamilian et al. [22], 2017	Cholecalciferol Cholecalciferol Placebo	SG low dose SG high dose PG	N/A	N/A	N/A	N/A	3.2 ± 0.4 3.2 ± 0.4 3.0 ± 0.3	2.9 ± 0.6 2.7 ± 0.4 * 3.1 ± 0.7
Jamilian et al. [2], 2017	Cholecalciferol+Magnesium+Zinc+Calcium Placebo	SG PG	86.6 ± 6.9 90.0 ± 4.8	86.7 ± 7.5 91.1 ± 5.9	12.9 ± 4.4 11.2 ± 3.9	11.0 ± 4.6 11.6 ± 4.5	2.8 ± 0.9 2.5 ± 0.9	2.4 ± 1.1 2.6 ± 1.0
Karamali et al. [19], 2018	Cholecalciferol + calcium + vitamin K Placebo	SG PG	84.2 ± 6.9 83.6 ± 13.0	84.5 ± 6.7 88.6 ± 20.5	11.8 ± 4.7 10.4 ± 5.3	9.9 ± 3.7 ** 12.2 ± 6.1	2.4 ± 1.0 2.1 ± 1.0	2.0 ± 0.8 ** 2.5 ± 1.3
Maktabi et al. [23], 2017	Cholecalciferol Placebo	SG PG	91.0 ± 6.1 93.8 ± 7.8	87.8 ± 7.6 * 94.3 ± 9.8	9.6 ± 4.5 9.1 ± 7.3	8.2 ± 2.8 ** 11.7 ± 6.5	2.2 ± 1.1 2.1 ± 1.7	1.8 ± 0.6 ** 2.7 ± 1.6
Raja-Khan et al. [20], 2014	Cholecalciferol Placebo	SG PG	84.9 ± 9.5 83.7 ± 9.3	83.8 ± 8.0 77.6 ± 14.7	26.31 ± 9.6 27.1 ± 15.8	38.1 ± 37.6 28.7 ± 14.6	5.5 ± 1.8 5.8 ± 3.9	7.8 ± 7.4 5.7 ± 3.0

N/A: not available. SG: supplemented group. PG: placebo group. ^a^ In this study, additional supplementation with calcium alone was also tested. These results were not taken into account in this meta-analysis. ^b^ In this study, a second control group only took metformin. These results were not taken into account in this meta-analysis. * *p* < 0.05; ** *p* < 0.01; *** *p* < 0.001.
